# Surface Compression Alters in Vivo VisR Stiffness, Viscosity, and Anisotropy Measurements in Human Breast

**DOI:** 10.1109/ojuffc.2025.3631426

**Published:** 2025-11-11

**Authors:** ANNA V. PHILLIPS, CHERIE M. KUZMIAK, DOREEN STEED, CATERINA M. GALLIPPI

**Affiliations:** 1 Lampe Joint Department of Biomedical Engineering, University of North Carolina at Chapel Hill, North Carolina State University, Chapel Hill, NC 27514 USA; 2 Department of Radiology, University of North Carolina at Chapel Hill, Chapel Hill, NC 27514 USA

**Keywords:** Acoustic radiation force, ARFI, breast cancer, breast imaging, elastography, non- invasive Imaging, pre-loading, strain elastography, viscoelastic response

## Abstract

Viscoelastic response (VisR) ultrasound has been developed by our group to interrogate tissue stiffness and viscosity. VisR has several potential advantages for breast cancer diagnostic imaging such being non-invasive and low-cost. Because ultrasound can penetrate dense breasts more effectively than mammograms, it may improve the detection of malignant masses in women with dense breasts. VisR-based estimates of stiffness, viscosity, and anisotropy have been shown in our preliminary studies to discriminate malignant and benign breast lesions. However, a potential limitation of VisR could be dependence on tissue pre-loading from applied surface compression by the practitioner. We conducted an IRB-approved clinical study of 20 women with no known breast pathologies to assess the impact of compression on VisR measurements of peak displacement (PD), relative elasticity (RE), relative viscosity (RV), and degree of anisotropy (DoA). Participants were between the ages of 30–90, and 10/20 had mammographically dense breasts. We found that surface compression significantly affected measurements of PD, RE, and RV in breast tissue, *in vivo*. In particular, in women with dense breasts, stiffness (via PD and RE) increased significantly with applied compression. DoA of PD, RE, and RV increased, decreased, or stayed the same with compression. No significant difference was found in DoA with compression between the breast density groups. Based on these findings, we recommend that surface compression be standardized and monitored when using VisR for clinical breast imaging, especially in women with dense breasts. Further studies are needed to identify an optimal strain range for VisR measurement repeatability.

## I. INTRODUCTION

Ultrasound elastography is an emerging modality of interest for breast cancer diagnosis due to its low cost, lack of invasiveness, and potential to supplement mammography. Using elastography measurements alongside the conventional mammogram can improve accuracy and specificity in detecting malignant breast lesions, even outperforming MRI [1]. An additional advantage of ultrasound is that it can visualize masses that may be obscured in mammography, improving detection and diagnosis in women with dense breasts [2], [3]. The sensitivity of mammography alone for detecting lesions in dense breasts can be as low as 62% [4]. Detection of suspicious lesions and correct diagnosis of cancer in women with dense breasts are highly relevant problems as women with dense breasts make up about 45% of the screening population [5].

Our research group has developed Viscoelastic Response (VisR) ultrasound, an elastography technique that interrogates tissue stiffness and viscosity. VisR works by using two consecutive acoustic radiation force impulse (ARFI) excitations to induce tissue displacements. The displacements are tracked and fit to a 1D mass-spring-damper model to semi-quantitatively estimate stiffness and viscosity relative to the force amplitude applied [6]. VisR can also be used to estimate tissue anisotropy, or the difference in mechanical properties in different loading orientations. In transversely isotropic (TI) tissue like skeletal muscle, VisR measurements can be compared between the transverse and longitudinal orientations in order to estimate the “degree of anisotropy” (DoA) [7], [8], [9]. Breast tissue can also be modeled as TI, as TI material models have been shown to accurately estimate deformations in the breast [10], [11].

Stiffness, viscosity, and anisotropy are biomarkers correlated with breast tumor malignancy [12], [13], [14], [15]. Breast tissue is also highly heterogeneous, and VisR is capable of interrogating these properties with finer spatial sampling than shear wave-based elastography techniques. We have shown in preliminary studies that VisR-based measurements of stiffness, viscosity, and anisotropy in breast lesions and surrounding tissue can discriminate between malignant and benign cases [16], [17]. Given these capabilities, VisR shows promise for improving breast cancer diagnosis compared to mammography alone or existing elastography techniques.

However, a potential limitation of VisR that has not yet been studied is the impact of practitioner-applied compression on the stiffness, viscosity, and anisotropy estimates. In clinical breast ultrasound scans, it is common for sonographers to manually apply varying levels of surface compression in order to achieve the best visual discernment of expected image features. Compression can also be applied unintentionally simply because the transducer is handheld, or from physiological motion. This results in pre-loading of the tissue, which may affect the Young’s modulus and therefore elastography results. Compounding this problem, breast tissue has a large range of reported Young’s modulus values both across and within different tissue types, and between different measurement techniques [18]. Thus, to create clinically feasible protocols for breast cancer diagnosis using VisR, it is important to characterize the impact of applied compression on the measurements.

The mechanical behavior of breast tissue under compression is poroelastic. When tissue is pre-loaded with small (less than a few percent) strain, fluid is forced out, and the tissue enters an approximately linear regime where Young’s modulus and shear modulus are proportional to stress and strain. This is the ideal regime in which to make reproducible stiffness measurements [19]. However, in clinical practice, the amount of compression applied can easily exceed this regime, meaning the relationship between stress, strain, and stiffness would no longer be linear [18]. This implies that pre-loading from applied compression could be very impactful on VisR measurements, as well as those made by other elasticity imaging approaches.

In this pilot clinical study, we collected VisR measurements at three levels of compression in women with and without dense breasts, all of whom had no known breast pathologies. The measurements were repeated at multiple imaging angles in each patient to enable anisotropy analysis. We hypothesize that VisR measurements of stiffness and viscosity will increase with applied surface compression, and that VisR measurements of anisotropy will also change significantly.

## II. METHODOLOGY

### A. CLINICAL IMAGING

The VisR imaging study was conducted at the University of North Carolina at Chapel Hill (UNC) Hospitals under IRB approval (#24–0122). Informed consent was obtained from all participants. Participants were women between the ages of 30–90 with no history of disease or biopsy in either breast. Imaging took place on the right breast in the 9 o’clock position, and all images were acquired by a trained sonographer. A focal depth of 20 mm was used for VisR to interrogate the mammary tissue layer and maintain as much consistency as possible in the spatial distribution of the applied ARF. The mammary layer was chosen because it contains the mammary ducts and is where the vast majority of breast tumors form [20].

A Siemens S3000 scanner and 9L4 probe (Siemens Healthineers, Issaquah, WA) were used to implement the VisR sequence [8]. The VisR sequence consisted of two reference pulses, two ARFI excitations separated by 8 tracking pulses, and 43 additional tracking pulses. ARFI excitations were 300 cycles at 4.21 MHz, and tracking pulses were 2 cycles at 6.15 MHz with a pulse repetition frequency of 11.5 KHz. The ARFI excitations used F/1.5 focal configuration. Reference and tracking pulses used F/1.5 focal configuration on transmit and F/0.75 on receive. For each data acquisition, the VisR sequence was repeated in 40 evenly spaced lateral positions across a 2 cm field of view to acquire a 2D image.

**FIGURE 1.**
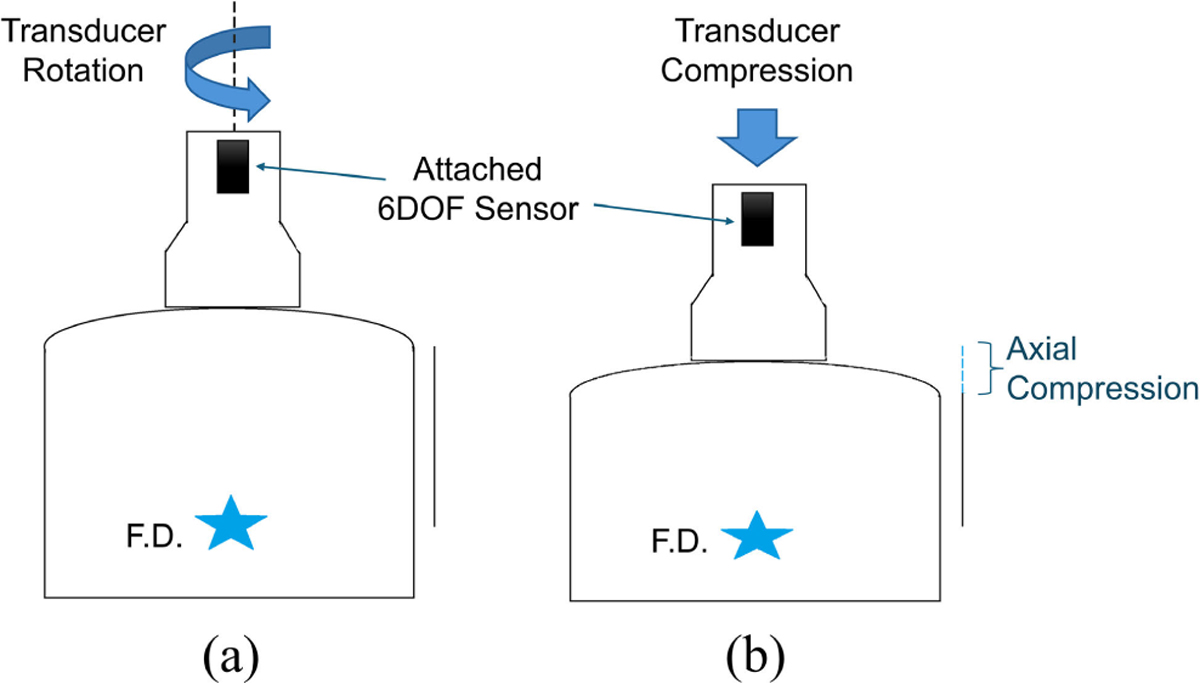
(a) The transducer was manually rotated about its center axis from 0–90° in steps of 30° to vary the position of the long-axis of the ARF excitation relative to the tissue axis of symmetry, enabling assessment of anisotropy. (b) Within each imaging angle, the transducer was also manually compressed in the axial direction to achieve 0–5 mm of compression relative to the focal depth (FD).

During imaging, VisR measurements were obtained at three clinically feasible levels of axial compression: 0, 2, and 5 mm. These compression levels correspond to approximately 0%, 10%, and 25% strain relative to the 20 mm focal depth, if assuming the stress from the compression is distributed uniformly in the tissue. The transducer was initially placed with the lightest possible contact to assume zero compression, then the transducer was pressed into the surface of the skin by the sonographer to obtain data at the higher compression levels, as shown in Figure 1. VisR measurements were taken at all three compression levels at four unique imaging angles, ranging from 0–90° in 30° increments. Transducer position was monitored and adjusted in real-time using a 3D Guidance Trakstar 6DOF sensor (Northern Digital Inc, Waterloo, Ontario, Canada).

As shown in Figure 1, the four imaging angles were achieved by rotating the transducer about its center axis from the initial position. We assume that the tissue is TI and has an axis of symmetry (AoS) parallel to the skin surface. Due to the asymmetry between the elevational and lateral dimensions of the transducer focus, each imaging angle has differing alignment between the tissue’s AoS and the ARF excitations for VisR. Each imaging angle then interrogates a different combination of the longitudinal and transverse mechanical moduli. Because of this, measurements of stiffness and viscosity vary over a 180° period in a TI material. This variation has previously been modeled as a sinusoid with the extrema of the sine wave representing the longitudinal and transverse moduli [21], [22]. However, an ellipse model is more accurate, especially in the case of high anisotropy [23]. Since the alignment of the material AoS in the patients’ breast tissue was not known a priori, VisR acquisitions were taken at 0°, 30°, 60°, and 90°. This range is guaranteed to span a half-period of variation with Nyquist sampling and therefore fully characterize the ellipse. The degree of anisotropy (DoA) of the tissue, representing the ratio between the dominant and non-dominant mechanical moduli, can then be estimated as the ratio of major to minor axis lengths of the ellipse.

### B. DATA PROCESSING

Tissue displacement from the VisR sequence was tracked with normalized cross-correlation using a 376-*μ*m kernel length, 80-*μ*m search region, and interpolation factor of 4. Peak displacement (PD), an estimate of tissue compliance, was calculated from the maximum value of tissue displacement over time. The displacement data was also fit to a 1D mass-spring-damper model to solve for the VisR parameters relative elasticity (RE) and relative viscosity (RV) [6]. To help ensure that the tissue being evaluated in each VisR acquisition had comparable forcing conditions, only PD, RE, and RV within 3 mm axially from the focal depth and 3 mm laterally from the center of the image were considered in our analyses.

To estimate the DoA of the breast tissue, PD, RE, and RV values across the four imaging angles were fit to an ellipse using least-squares minimization. DoA was calculated as ratio of major axis length to minor axis length of the ellipse fitted to the VisR data. DoA of PD, RE, and RV was compared between the three compression levels for each patient and between the dense and non-dense breast groups. Additionally, the percentage change in PD, RE, and RV was calculated between each compression level for each angle. Since PD, RE, and RV are relative to the acoustic force applied, percentage change is a normalized metric that can be compared between patients assuming force remains constant within a patient as compression changes.

For comparisons of PD, RE, and RV between compression levels within each patient, the Kruskal-Wallis test was used. For comparisons of DoA, change in DoA, and change in PD, RE, and RV between the different compression levels, the Wilcoxon Rank Sum test was used. Wilcoxon Rank Sum was also used for all comparisons between the dense and non-dense breast groups and medical history groups.

## III. RESULTS

Table 1 shows the BI-RADS density rating, as assessed by a breast radiologist, for each subject. There were 10 women with non-dense and 10 women with dense breasts. All of the subjects were rated B or C, which are the most common ratings in the screening population [5]. Age, history of pregnancy and breastfeeding, and family history of breast cancer are also shown.

**FIGURE 2.**
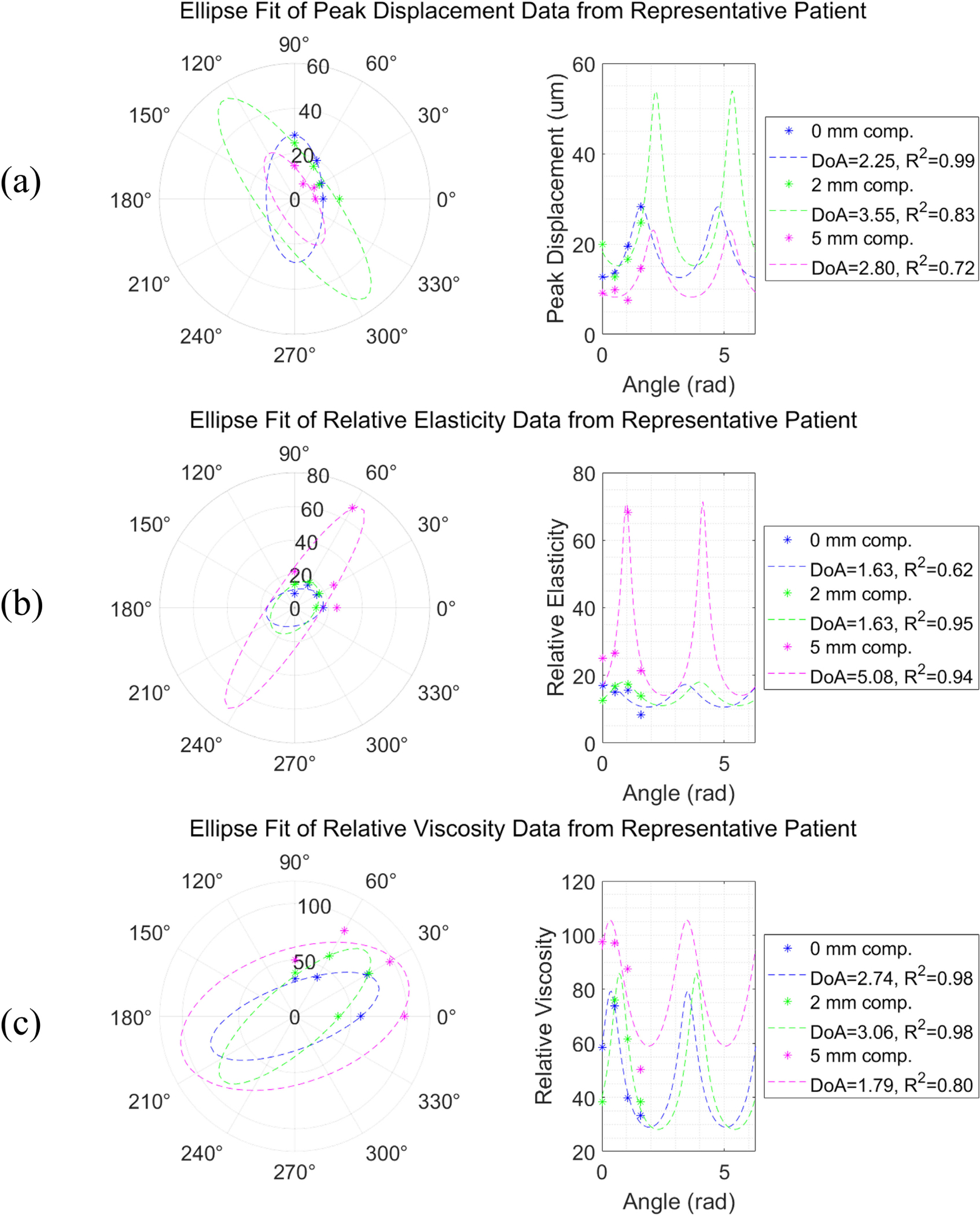
Peak displacement (PD), relative elasticity (RE), and relative viscosity (RV) varied elliptically in the breast tissue of this representative patient between 0–90° imaging angles. Degree of anisotropy (DoA), the ratio of major to minor axis length of the ellipse, is shown on each plot.

Figure 2 demonstrates the elliptical variation of PD, RE, and RV values across measurement angles at 0, 2, and 5 mm of compression in a representative patient from the study. As expected, PD has inverted phase to RE as PD represents compliance and RE represents stiffness. RE and RV, representing stiffness and viscosity, vary together in phase as expected for soft tissue. The breast tissue demonstrates anisotropy, as DoA is > 1 in PD, RE, and RV. In this patient, the elliptical approximation for anisotropy was relatively accurate, given the high R-squared values (avg=0.9). Supplemental Table 2 shows R-squared values for all participants.

Between the 0, 2, and 5 mm compression levels at each angle, PD, RE, and RV values changed significantly in this patient. We expected that with the amount of pre-loading applied, the tissue may not be in the linear stress-strain relationship regime, and indeed, PD, RE, and RV did not appear to change linearly with compression. DoA also changed with compression, but not monotonically.

Statistical test results for PD, RE, and RV values differing with compression in each patient using Kruskal-Wallis tests are shown in Supplemental Table 3, with alpha level adjusted using the Bonferroni correction for multiple comparisons. In general, PD, RE, and RV values within each angle were strongly significantly different with compression for all patients. Difference in DoA between compression levels could not be statistically compared in this manner for individual patients since DoA is a single value rather than a distribution.

**FIGURE 3.**
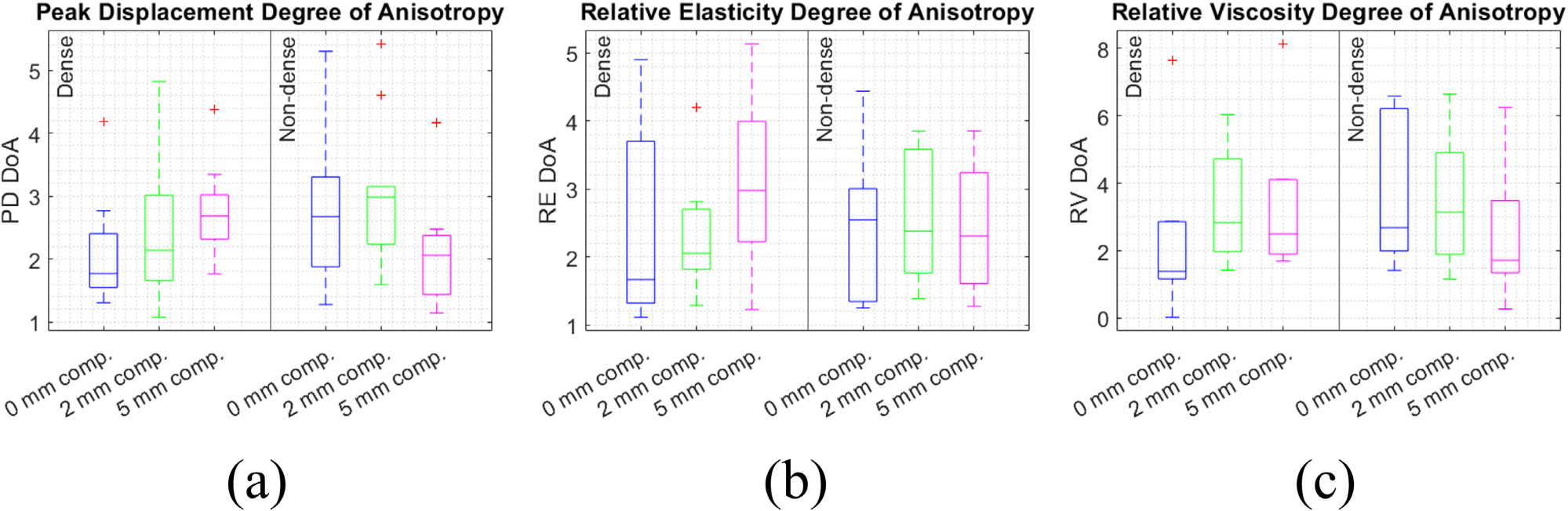
Degree of anisotropy (DoA) of peak displacement (a), relative elasticity (b), and relative viscosity (c) under compression. DoA compared between the dense (left) and non-dense (right) breast groups, and for 0, 2, 5 mm compression.

The DoA of PD, RE, and RV at each compression level are shown for all patients, separated by dense and non-dense breast groups, in Figure 3. The distribution of DoA at each compression level did not significantly differ between the breast density groups (Wilcoxon Rank Sum test). Despite per-angle PD, RE, and RV values changing significantly with compression within each patient, DoA of PD, RE, and RV did not significantly differ between the compression levels in all patients.

Median DoA for all strain levels and both density groups was >1 for PD, RE, and RV, indicating the breast tissue of the subjects is anisotropic in general. This range of DoA values agrees with values from our previous pilot study in breast cancer patients, in which DoA in non-tumor mammary tissue was mostly between 2–4 [22]. There is little other reporting on expected DoA values in the breast, but the ratio of shear wave speed between radial and anti-radial directions has been reported as ~1.3 [24].

**FIGURE 4.**
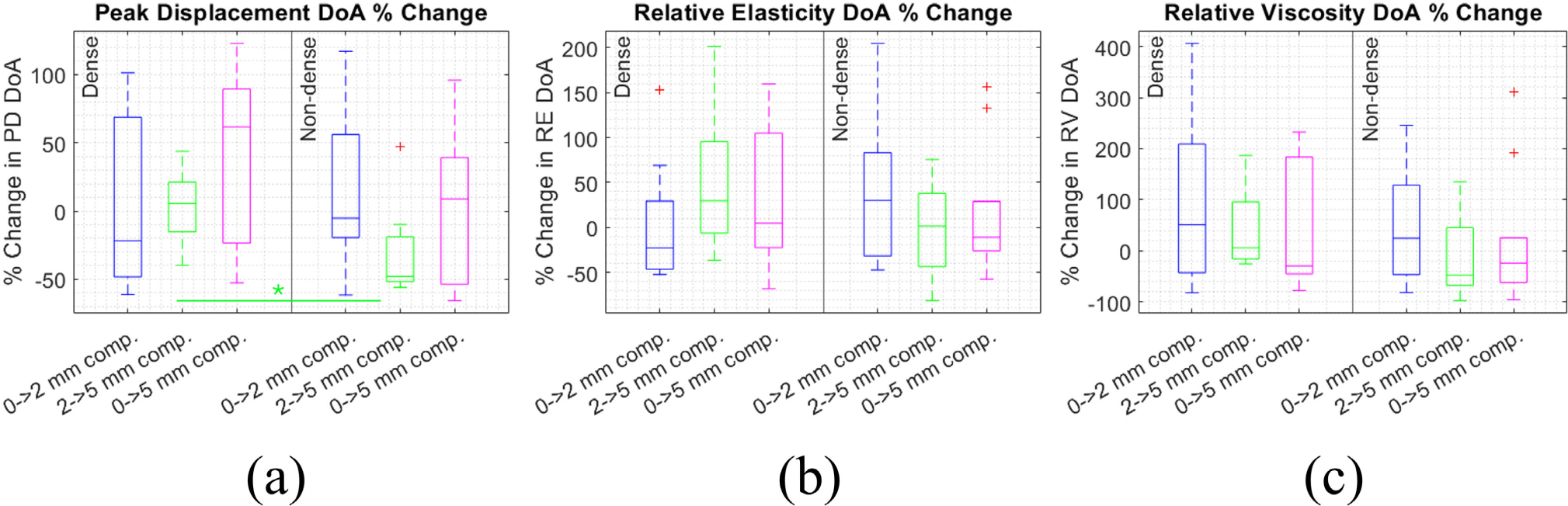
Percent change in degree of anisotropy (DoA) of peak displacement (a), relative elasticity (b), and relative viscosity (c) under compression. Percent change compared between the dense (left) and non-dense (right) breast groups, and from 0–2, 2–5, and 0–5 mm compression.

Figure 4 shows within-patient percentage change in the DoA of PD, RE, and RV between the 0–2, 2–5, and 0–5 mm compression levels. Medians range from −48% to 61% for PD, −23% to 30% for RE, and −48% to 51% for RV, indicating compression can cause large changes in VisR DoA, especially for RV which had the largest inter-quartile ranges. Interestingly, DoA of PD, RE, and RV increased with compression for some patients but decreased for others. Between 2–5 mm compression, percent change in PD DoA was significantly lower in the non-dense group. Otherwise, the difference in DoA change with compression was not significant between the dense and non-dense groups, nor between the compression levels (Wilcoxon Rank Sum test).

**FIGURE 5.**
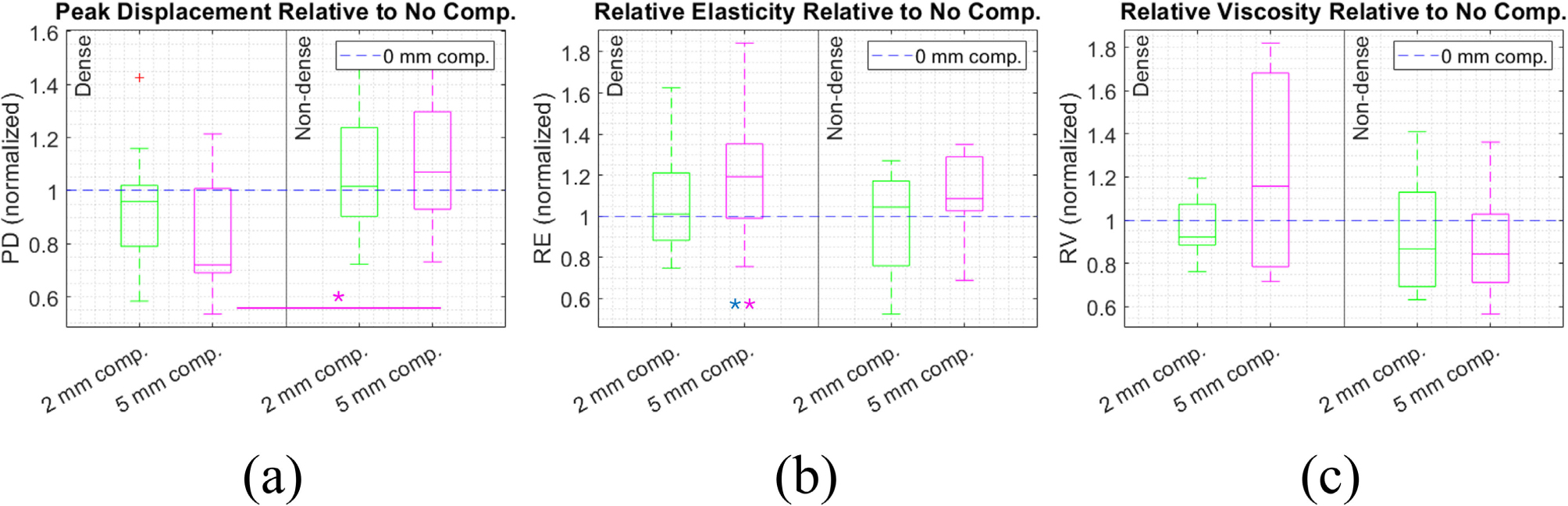
Peak displacement (a), relative elasticity (b), and relative viscosity (c) under compression, averaged across angles and normalized by zero compression value. Values compared between the dense (left) and non-dense (right) breast groups, and for 0, 2, 5 mm compression.

Figure 5 shows PD, RE, and RV within each patient at 2 and 5 mm of compression, averaged between the four imaging angles. Since PD, RE, and RV are relative to applied force and different tissue compositions could affect the ARF deposited at the focal depth, values are normalized relative to the 0 mm compression value. At 5 mm of compression, PD is significantly lower in the dense breast group. Additionally, RE was significantly reduced at 5 mm compression compared to at 0 mm compression in the dense breast group. RV did not have a significant trend with compression (Wilcoxon Rank Sum test).

**FIGURE 6.**
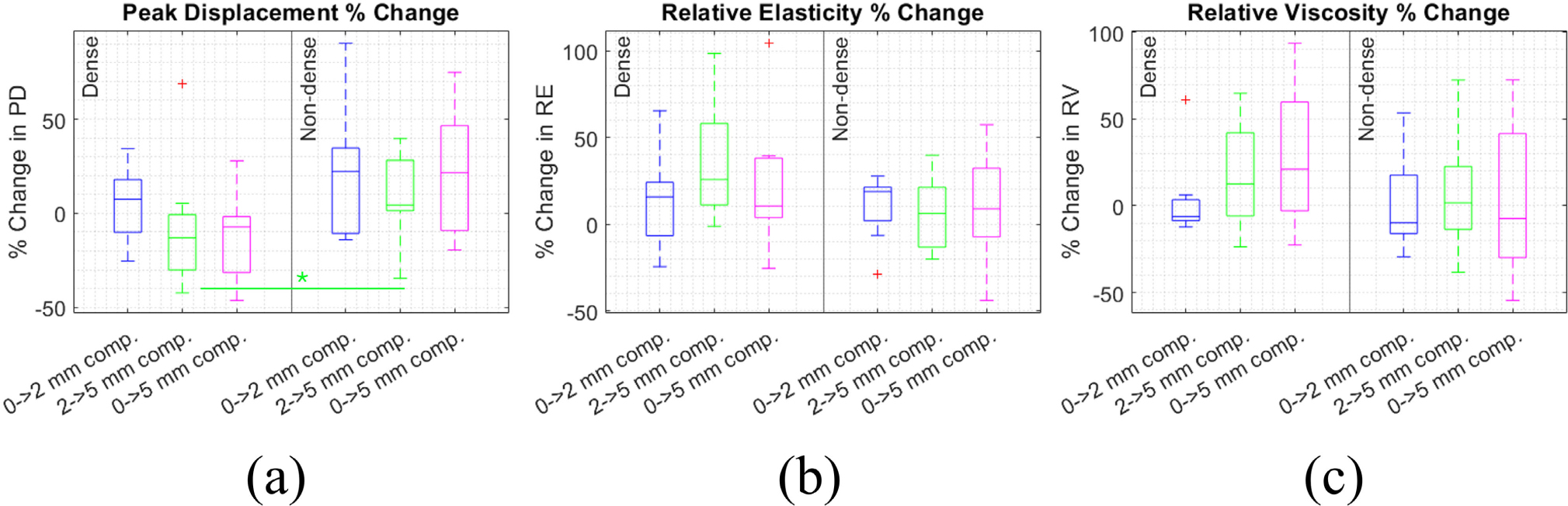
Percent change in peak displacement (a), relative elasticity (b), and relative viscosity (c) under compression. Percent change compared between the dense (left) and non-dense (right) breast groups, and from 0–2, 2–5, and 0–5 mm compression.

Furthermore, Figure 6 shows percentage change in of PD, RE, and RV within each patient between the 0–2, 2–5, and 0–5 mm compression levels, also averaged over angle. Median percent change ranged from −13% to 22% for PD, 6.2% to 26% for RE, and −9.8% to 21% for RV. This corroborates the result that PD, RE, and RV were altered between compression levels in individual subjects. Like DoA, PD, RE, and RV increased in some patients and decreased in others under compression. The ranges of percent change values for PD, RE, and RV are smaller than for DoA. Percent change in PD between 2–5 mm compression was significantly lower in the dense breast group. Otherwise, differences in percent change were not significant between the dense and non-dense breast groups, between the different strain ranges, nor between PD, RE, and RV (Wilcoxon Rank Sum test).

**FIGURE 7.**
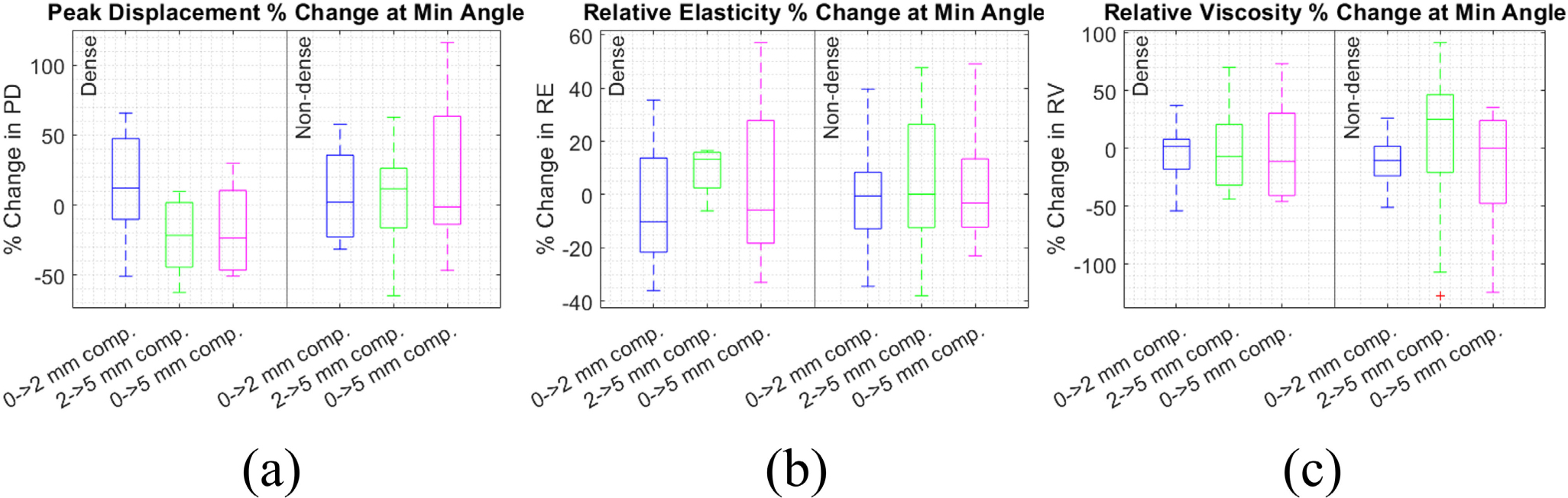
Percent change in peak displacement (a), relative elasticity (b), and relative viscosity (c) under compression in the imaging angle with smallest value. Percent change compared between the dense (left) and non-dense (right) breast groups, and from 0–2, 2–5, and 0–5 mm compression.

**FIGURE 8.**
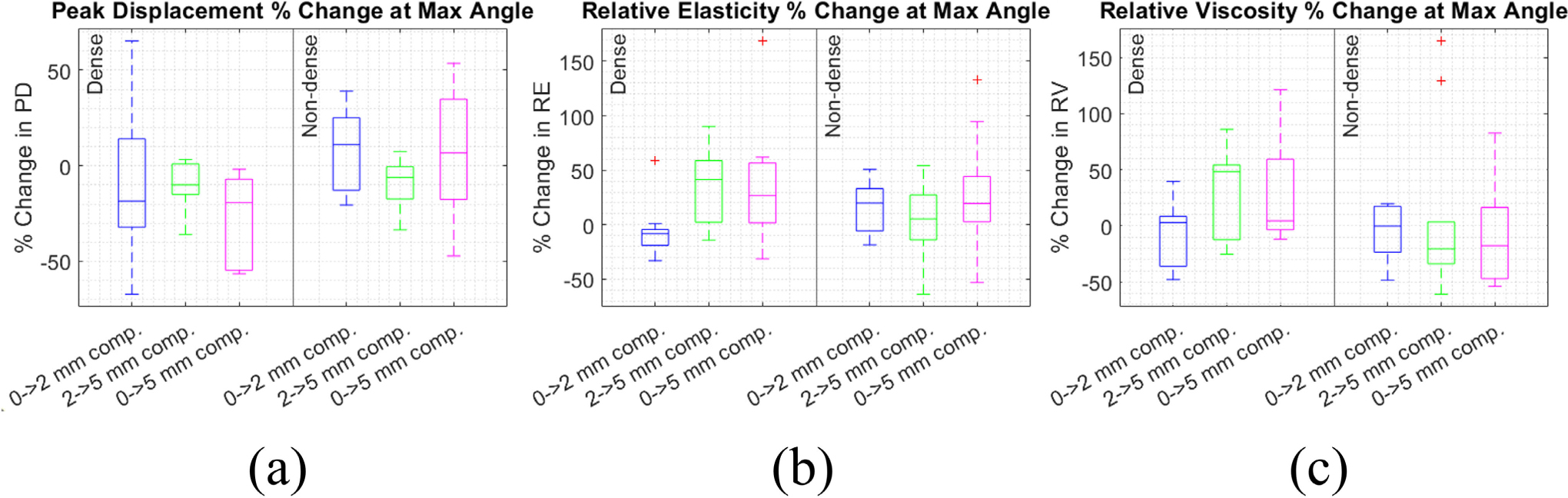
Percent change in peak displacement (a), relative elasticity (b), and relative viscosity (c) under compression in the imaging angle with largest value. Percent change compared between the dense (left) and non-dense (right) breast groups, and from 0–2, 2–5, and 0–5 mm compression.

We also assessed percentage change in PD, RE, and RV with compression for only the angles where PD, RE, and RV were minimized (Figure 7) and maximized (Figure 8) in each subject. As shown in Figure 7, median percent change at the angle at which minimum was achieved (which we henceforth refer to as the “minimum angle”) varied from −22% to 14% for PD, −10% to 13% for RE, and −10% to 25% for RV. The changes in PD, RE, and RV at the minimum angle were not significantly different than they were across all angles (Figure 6). As shown in Figure 8, median percent change at the angle at which maximum was achieved (which we henceforth refer to as the “maximum angle”) ranged from −19% to 11% for PD, −8.2% to 42% for RE, and −20% to 48% for RV. The changes in PD, RE, and RV at the maximum angle were also not significantly different than they were across all angles (Figure 6), nor from the changes at the minimum angle.

**FIGURE 9.**
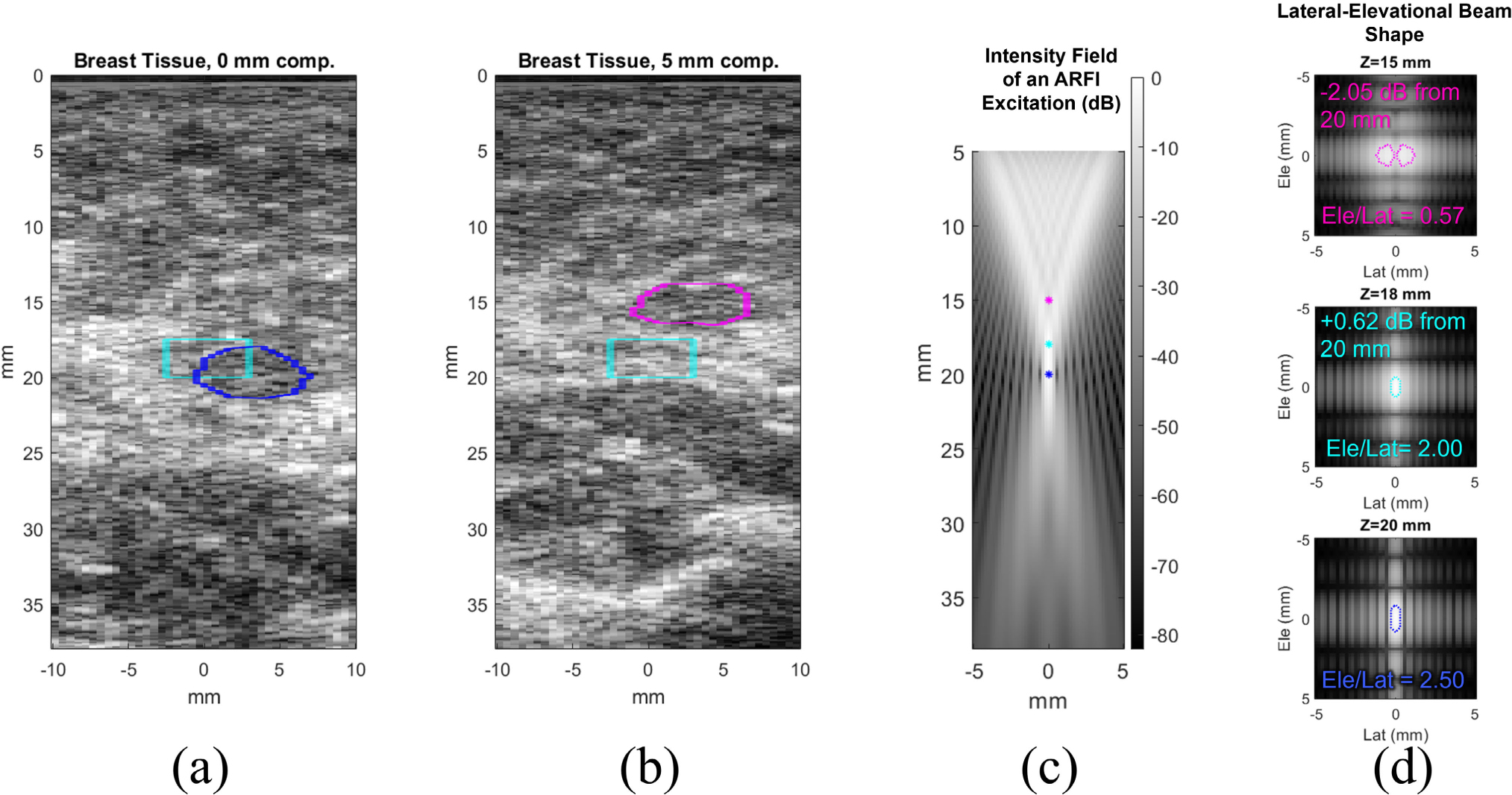
The shift in tissue axial position between 0 and 5 mm compression are shown in a representative patient in (a) and (b). The cyan ROI is fixed around the ARFI focal depth, while blue and magenta ROI capture the same tissue feature at the two different compression levels. With a fixed focus at 20 mm, ARFI intensity is different in the three ROI (c). The lateral-elevational beam shape also changes when ROI are selected at different depths (d).

Figure 9 demonstrates the limitations of two different approaches for placing a region of interest (ROI) in the images for data analysis. The cyan ROI in (a) and (b) represents an automated approach, where all tissue < 3 mm of the center of the image laterally and 3 mm above the focus axially is included. This approach ensures that beam shape and force applied is as similar as possible in the ROI between acquisitions at different compression levels. However, doing so results in different tissue entering the region of interest when compression is applied. As shown in (a) and (b), the same echogenic layer of fibroglandular tissue changes axial depth between 0 and 5 mm compression. To ensure that the same tissue is interrogated between different compression levels, the ROI can be adaptively placed around tissue features. This is demonstrated by the blue ROI in (a) and magenta ROI in (b). With this approach, the axial and lateral limits of the ROI could vary between acquisitions, resulting in different forcing conditions.

The differences in focal characteristics of the ARFI excitations when adjusting the ROI to different depths are quantified in Figure 9 (c) and (d). Since the focal depth of the ARF is 20 mm, changing the depth of the ROI causes the acoustic intensity to differ. Moving the ROI up by 5 mm causes an intensity loss of 2.05 dB, which could affect measured PD, RE, and RV values. If the ROI is at the same depth at each imaging angle, theoretically PD, RE, and RV should be scaled by the same amount so that DoA is unaffected. However, in many cases the ROI depth could also differ by angle. Further, the lateral-elevational shape of the beam, which is relevant for measuring anisotropy, changes. Moving 2 mm up from the focal depth causes the ratio of elevational to lateral beam width to decrease from 2.5 to 2.0, and moving 5 mm up decreases this ratio to just 0.57. This means that shifting the ROI by a few mm in an image could greatly alter measured DoA. To minimize these limitations, we adapted ROI in the study to capture the same tissue between compression levels, but also cropped the ROI to within <3 mm of the focal region.

## IV. DISCUSSION

This study emulated clinical breast ultrasound imaging conditions by using manual transducer placement, and clinically feasible levels of axial compression in the range of 0–5 mm. The DoA using all three VisR metrics was not significantly different between the dense and non-dense breast groups nor between the different strain levels. Percent change in DoA also was not significantly different between the dense and non-dense groups, other than PD-derived DoA between 2–5 mm compression. Within individual subjects, DoA sometimes increased, sometimes decreased, and sometimes did not change with compression.

We found that within each patient, PD, RE, and RV statistically significantly differed between the three strain levels. The median percent changes in RE and RV from 0–2 mm, 2–5 mm, and 0–5 mm were largely positive in the dense group. For RE and RV, this may support our hypothesis that applied compression would increase stiffness and viscosity due to the poro-elasticity of the material. However in the non-dense group, RE and RV percent changes were on average closer to zero. Furthermore, percent change in PD from 2–5 mm compression was significantly lower in the dense group. PD values at 5 mm compression, normalized by zero compression value, were significantly lower in the dense group than non-dense. RE was also significantly increased at 5 mm compression relative to zero in the dense group. These results indicate that tissue stiffness increased significantly more under compression in the dense breast patients.

Considering only the angles for which PD, RE, and RV were maximized or minimized in each subject, percent changes in PD, RE, and RV with compression were not significantly different than for all angles. Results from individual subjects suggest that PD, RE, and RV measurements can be affected by compression more in certain orientations, but there is no conclusive evidence in the entire dataset that the transverse or longitudinal modulus is affected more on average. Based on the structure of the breast, we would expect the longitudinal modulus in the TI material model to represent the radial orientation and the transverse modulus to represent the anti-radial orientation [15]. Furthermore, PD, RE, RV, or DoA of the three metrics did not change significantly differently between 0–2 mm compression and 2–5 mm compression in the entire dataset. There is insufficient evidence to say that a particular compression amount or imaging angle results in more consistent VisR measurements in the breast.

The range of PD, RE, and RV percent changes with compression was smaller than the range of DoA percent changes with compression. The large variation in both DoA values and change in DoA with compression was likely due to several factors like variations in tissue composition and breast geometry. Our definition of DoA also assumes that the material axis of symmetry is parallel to the skin surface, which may not be correct. If the AoS is not parallel to the skin surface, VisR is not interrogating only shear behavior but also compressive behavior and may be inaccurate. Another potential reason for the large range in DoA is that DoA is calculated based on an ellipse fit to the data at four angles. Changes in stiffness and viscosity at any of the imaging angles could impact DoA, especially if some angles have larger and some have smaller changes.

We found that interrogating a consistent region of tissue with the same applied force between different strain levels and imaging angles was challenging. Adjusting the ROI of VisR measurements to match tissue features across different images can mean that the tissue has different forcing conditions in each image. If applied force is not consistent, comparisons of PD, RE, and RV may not be relevant. Therefore, in this study changes in VisR measurements between compression levels could be attributed to changing mechanical properties in the tissue, effects from altered force propagation, or differing ROI selection. Because of this, it is important to avoid variations in surface compression during clinical data collection as much as possible. One potential solution for this issue would be to do an initial B-Mode sweep of the tissue at different compression levels and angles, and adaptively adjusting the ROI to maximize signal correlation of tissue features between them. Then, the VisR sequence could be executed with the focal depth adjusted to the ROI at each angle. However, the differing focal depth could affect applied ARF and thus PD, RE, and RV values. Another potential solution would be to use a larger ROI which incorporates more tissue. A larger ROI would be more likely to capture the same tissue features between acquisitions while maintaining similar forcing conditions. However, it would also be averaging over more area which reduces spatial sampling and the ability to understand heterogeneities in the tissue. Lastly, it could be an improvement to use B-Mode to estimate strain in the tissue, and acquire VisR data when and where strain is minimized.

Stiffness and viscosity based on VisR PD, RE, and RV did not increase with compression in every subject as expected for a poroelastic material. One reason for this is that VisR only uses a 1D axial mass-spring-damper model to assess mechanical properties from tissue displacement, but breast geometry, and its response to ARF excitations, is more complex than the mass-spring-damper model predicts. Further, patient and sonographer motion might have further confounded the derivation of mechanical property from observed displacements. Finally, 2D imaging was implemented using a linear array, so tissue motion was averaged over the 2–4mm resolution volume in the elevational direction. It is possible that at different levels of compression, different tissue features in elevation that were invisible in B-mode were contributing to mechanical property measurements, further confounding outcomes. This effect could be impactful in diagnostic imaging of breast lesions, as they can also have complex, heterogeneous geometry.

The main limitation of this study is small sample size (10 women with dense breasts and 10 without). More subjects, especially women representing BI-RADS A and D densities, could further establish the impact of compression on the measurements and optimal conditions for repeatable measurements. The breast density of women in the study was entirely BI-RADS B-C, but we would expect the most significant differences in mechanical behavior between women on the extremes of the scale, i.e. A or D ratings. Higher breast density, especially a D BI-RADS rating, is correlated with higher stiffness, more anisotropic collagen organization in the mammary tissue extracellular matrix, and higher breast cancer risk [25]. Furthermore, a larger sample size would allow better assessment of other variables affecting breast mechanical behavior, such as age and reproductive history. No significant differences in PD, RE, RV, and DoA were found based on history of pregnancy, breastfeeding, or breast cancer in the family, but this could be due to a lack of statistical power.

Another limitation is that within each patient, the stress and strain resulting from surface compression differs. The propagation of stress to the imaging focal depth from the surface may be non-uniform, making it difficult to compare mechanical behavior of tissue between patients. The stress propagation may also be affected by breast structure and density. Mechanical loading could be quantified using a surface pressure sensor combined with image-based strain estimation, however this is outside the scope of this work. The compression in this study was meant to replicate variations in applied pressure that occur during routine clinical breast imaging.

Finally, the 1D mass-spring-damper model used in VisR is an approximation and estimates of tissue mechanical properties may be improved by a more complex model. Incorporating nonlinear elasticity or 3D connectivity might improve accuracy. However, it is difficult to translate computational models to *in vivo* behavior in breast tissue because it is highly heterogeneous and variable between individuals.

## V. CONCLUSION

We assessed the impact of manually applied surface compression on VisR measurements of stiffness, viscosity, and anisotropy via PD, RE, RV, and DoA in the mammary tissue of 20 women with no history of breast disease. We found no significant trends in DoA between women with and without dense breasts, nor between different compression levels. Percent change in DoA with compression also did not differ between women with and without dense breasts, other than for PD-derived DoA. However, DoA increased or decreased within individual subjects when compression was applied.

We also found that PD, RE, and RV were strongly altered when compression was applied. In women with dense breasts, tissue stiffness measured with PD and RE significantly increased with compression compared to the non-dense group. No significant difference was found in RV under compression between the dense and non-dense groups. Percent change in PD, RE, and RV with compression also was not significantly different between the “minimum” angle, “maximum” angle, and all-angle average. Based on these results, we conclude that VisR results are impacted by applied compression. As the compression range used in this work was in the clinically relevant range for a sonographer, compression should be standardized and monitored when taking VisR measurements to avoid this confounding factor. We did not find evidence to support that the 0, 2, or 5 mm range is better for measurement consistency, but limiting measurements to a constant compression level and similar focal depth would ensure similar pre-loading within a particular patient and region of tissue.

Future work could entail more subjects in order to better assess the impact of breast density on the four-point BI-RADS scale on VisR measurements and measurement consistency under compression. Other demographic and medical history factors such as BMI and parity, which are risk factors for breast cancer, could also be assessed [26], [27], [28]. Based on the variation in R-squared values between patients when fitting the VisR data across imaging angles to the TI tissue model, more complex models aside from transversely isotropic could also be considered. A larger range and finer sampling of compression levels and imaging angles would enable more complex models to be tested, at the expense of slower imaging exam times. The ultimate goal of such work would be to develop a replicable diagnostic protocol for breast cancer diagnosis and treatment monitoring using VisR exams.

## Figures and Tables

**TABLE 1.** Breast density ratings and medical history of study participants.

Patient #	BI-RADS Density	Age	History of Pregnancy	History of Breast-feeding	Family History of Breast Cancer

1	B	72	Yes	No	Yes
2	B	68	Yes	Yes	Yes
3	B	72	Yes	Yes	Unknown
4	C	64	Yes	Yes	No
5	C	50	Yes	Yes	No
6	B	60	Yes	Unknown	Unknown
7	B	65	Yes	Yes	No
8	B	61	Yes	Yes	Yes
9	B	60	No	No	Yes
10	C	67	Yes	Yes	Yes
11	C	40	Yes	Yes	No
12	B	47	Yes	No	Yes
13	B	50	Yes	Yes	No
14	C	50	No	No	Yes
15	C	44	No	No	Yes
16	B	52	Yes	Yes	No
17	C	48	Yes	Yes	No
18	C	46	Yes	Yes	Yes
19	C	66	Yes	Yes	No
20	C	60	Yes	Yes	Yes

The BI-RADS breast density scale ranges from A (almost entirely fatty) to D (almost entirely dense). A-B is considered non-dense breasts and C-D is considered dense breasts in clinical practice.

**TABLE 2.** R-squared value for data across angles fit to ellipse for PD, RE, and RV at 0–5 mm compression.

Patient	PD 0 mm	PD 2 mm	PD 5 mm	RE 0 mm	RE 2 mm	RE 5 mm	RV 0 mm	RV 2 mm	RV 5 mm
1	0.65	0.28	0.78	0.77	0.68	0.23	0.92	0.98	0.98
2	0.92	0.93	0.23	0.63	0.89	0.84	0.96	0.95	0.42
3	0.12	0.92	0.96	0.49	0.93	0.85	0.49	0.87	0.57
4	0.95	0.75	0.78	0.09	0.41	0.41	0.92	0.77	0.84
5	0.44	0.05	0.70	0.89	0.68	0.94	0.77	0.83	0.97
6	0.96	0.57	0.65	0.09	0.77	0.58	0.89	0.70	0.58
7	0.49	0.59	0.61	0.74	0.56	0.54	0.14	0.63	0.90
8	0.82	0.99	0.93	0.91	0.92	0.98	0.69	0.93	0.88
9	0.36	0.97	0.46	0.58	0.79	0.83	0.22	0.70	0.29
10	0.97	0.55	0.41	0.87	0.88	0.94	0.98	0.88	0.83
11	0.97	0.98	0.83	0.94	0.69	0.92	0.87	0.90	0.91
12	0.99	0.80	0.86	0.93	0.95	0.96	0.61	0.99	0.47
13	0.75	0.92	0.88	0.69	0.66	0.92	0.39	0.84	0.81
14	0.82	0.66	0.68	0.96	0.57	0.35	0.70	0.96	0.52
15	0.95	0.83	0.92	1.00	0.98	0.96	0.79	0.98	0.93
16	0.99	0.35	0.01	0.45	0.06	0.26	0.91	0.70	0.93
17	0.18	0.97	0.45	0.58	0.87	0.98	0.97	0.55	0.54
18	0.98	0.81	0.50	0.59	0.91	0.91	0.99	0.98	0.95
19	0.89	0.94	0.98	0.90	0.97	0.98	0.39	0.97	0.92
20	0.67	0.97	0.97	0.87	0.98	0.86	0.79	0.98	0.74
Avg.	0.89	0.81	0.70	0.74	0.79	0.91	0.79	0.88	0.84

**TABLE 3.** **Results from Kruskal-Wallis test between PD, RE, and RV values at 0, 2, and 5 mm compression and at each imaging angle for the patients. Alpha level is based on Bonferroni correction for multiple comparisons (***α* = 0.05/(12 * 20) = 2.08E – 4).

Patient	PD 0°	PD 30°	PD 60°	PD 90°	RE 0°	RE 30°	RE 60°	RE 90°	RV 0°	RV 30°	RV 60°	RV 90°
1	*p* ≪ *α*	*p* ≪ *α*	*p* ≪ *α*	*p* ≪ *α*	*p* ≪ *α*	*p* ≪ *α*	*p* ≪ *α*	*p* ≪ *α*	*p* ≪ *α*	*p* ≪ *α*	*p* ≪ *α*	*p* ≪ *α*
2	*p* ≪ *α*	*p* ≪ *α*	*p* ≪ *α*	*p* ≪ *α*	*p* ≪ *α*	*p* ≪ *α*	*p* ≪ *α*	*p* ≪ *α*	*p* ≪ *α*	*p* ≪ *α*	*p* ≪ *α*	*p* ≪ *α*
3	*p* ≪ *α*	*p* ≪ *α*	*p* ≪ *α*	*p* ≪ *α*	*p* ≪ *α*	*p* ≪ *α*	*p* ≪ *α*	*p* ≪ *α*	*p* ≪ *α*	*p* ≪ *α*	*p* ≪ *α*	*p* ≪ *α*
4	*p* ≪ *α*	*p* ≪ *α*	*p* ≪ *α*	*p* ≪ *α*	*p* ≪ *α*	*p* ≪ *α*	*p* ≪ *α*	*p* ≪ *α*	*p* ≪ *α*	*p* ≪ *α*	*p* ≪ *α*	*p* ≪ *α*
5	*p* ≪ *α*	*p* ≪ *α*	*p* ≪ *α*	*p* ≪ *α*	*p* ≪ *α*	*p* ≪ *α*	*p* ≪ *α*	*p* ≪ *α*	*p* ≪ *α*	*p* ≪ *α*	*p* ≪ *α*	*p* ≪ *α*
6	*p* ≪ *α*	*p* ≪ *α*	*p* ≪ *α*	*p* ≪ *α*	*p* ≪ *α*	*p* ≪ *α*	*p* ≪ *α*	*p* ≪ *α*	*p* ≪ *α*	*p* ≪ *α*	p=6.4E-04	*p* ≪ *α*
7	*p* ≪ *α*	*p* ≪ *α*	*p* ≪ *α*	*p* ≪ *α*	*p* ≪ *α*	*p* ≪ *α*	*p* ≪ *α*	*p* ≪ *α*	*p* ≪ *α*	*p* ≪ *α*	*p* ≪ *α*	*p* ≪ *α*
8	*p* ≪ *α*	*p* ≪ *α*	*p* ≪ *α*	*p* ≪ *α*	*p* ≪ *α*	*p* ≪ *α*	*p* ≪ *α*	*p* ≪ *α*	*p* ≪ *α*	*p* ≪ *α*	*p* ≪ *α*	*p* ≪ *α*
9	*p* ≪ *α*	*p* ≪ *α*	*p* ≪ *α*	*p* ≪ *α*	*p* ≪ *α*	*p* ≪ *α*	*p* ≪ *α*	*p* ≪ *α*	*p* ≪ *α*	*p* ≪ *α*	*p* ≪ *α*	*p* ≪ *α*
10	*p* ≪ *α*	*p* ≪ *α*	*p* ≪ *α*	*p* ≪ *α*	*p* ≪ *α*	*p* ≪ *α*	*p* ≪ *α*	*p* ≪ *α*	p=2.7E-04	*p* ≪ *α*	*p* ≪ *α*	*p* ≪ *α*
11	*p* ≪ *α*	*p* ≪ *α*	*p* ≪ *α*	*p* ≪ *α*	*p* ≪ *α*	*p* ≪ *α*	*p* ≪ *α*	*p* ≪ *α*	*p* ≪ *α*	*p* ≪ *α*	*p* ≪ *α*	*p* ≪ *α*
12	*p* ≪ *α*	*p* ≪ *α*	*p* ≪ *α*	*p* ≪ *α*	*p* ≪ *α*	*p* ≪ *α*	*p* ≪ *α*	*p* ≪ *α*	*p* ≪ *α*	*p* ≪ *α*	*p* ≪ *α*	*p* ≪ *α*
13	*p* ≪ *α*	*p* ≪ *α*	*p* ≪ *α*	*p* ≪ *α*	*p* ≪ *α*	*p* ≪ *α*	*p* ≪ *α*	*p* ≪ *α*	*p* ≪ *α*	*p* ≪ *α*	*p* ≪ *α*	*p* ≪ *α*
14	*p* ≪ *α*	*p* ≪ *α*	*p* ≪ *α*	*p* ≪ *α*	*p* ≪ *α*	*p* ≪ *α*	*p* ≪ *α*	*p* ≪ *α*	*p* ≪ *α*	*p* ≪ *α*	*p* ≪ *α*	*p* ≪ *α*
15	*p* ≪ *α*	*p* ≪ *α*	*p* ≪ *α*	*p* ≪ *α*	*p* ≪ *α*	*p* ≪ *α*	*p* ≪ *α*	*p* ≪ *α*	*p* ≪ *α*	*p* ≪ *α*	*p* ≪ *α*	*p* ≪ *α*
16	*p* ≪ *α*	*p* ≪ *α*	*p* ≪ *α*	*p* ≪ *α*	*p* ≪ *α*	p=4.6E-03	*p* ≪ *α*	*p* ≪ *α*	*p* ≪ *α*	*p* ≪ *α*	*p* ≪ *α*	*p* ≪ *α*
17	*p* ≪ *α*	*p* ≪ *α*	*p* ≪ *α*	*p* ≪ *α*	*p* ≪ *α*	*p* ≪ *α*	*p* ≪ *α*	*p* ≪ *α*	*p* ≪ *α*	p=2.8E-04	*p* ≪ *α*	*p* ≪ *α*
18	*p* ≪ *α*	*p* ≪ *α*	*p* ≪ *α*	*p* ≪ *α*	*p* ≪ *α*	*p* ≪ *α*	*p* ≪ *α*	*p* ≪ *α*	*p* ≪ *α*	*p* ≪ *α*	*p* ≪ *α*	*p* ≪ *α*
19	*p* ≪ *α*	*p* ≪ *α*	*p* ≪ *α*	*p* ≪ *α*	*p* ≪ *α*	*p* ≪ *α*	*p* ≪ *α*	*p* ≪ *α*	*p* ≪ *α*	*p* ≪ *α*	*p* ≪ *α*	*p* ≪ *α*
20	*p* ≪ *α*	*p* ≪ *α*	*p* ≪ *α*	*p* ≪ *α*	*p* ≪ *α*	*p* ≪ *α*	*p* ≪ *α*	*p* ≪ *α*	*p* ≪ *α*	*p* ≪ *α*	*p* ≪ *α*	*p* ≪ *α*

## APPENDIX

Table 2 shows the R-squared values for VisR data across the multiple imaging angles within each patient and strain level fit to an ellipse. Table 3 shows Kruskal-Wallis test results for difference in PD, RE, and RV within each patient and imaging angle between the three compression levels.
